# Recombinant lentivirus with enhanced expression of caudal-related homeobox protein 2 inhibits human colorectal cancer cell proliferation *in vitro*

**DOI:** 10.3892/mmr.2015.3594

**Published:** 2015-04-03

**Authors:** SAI HE, XUE-JUN SUN, JIAN-BAO ZHENG, JIE QI, NAN-ZHENG CHEN, WEI WANG, GUANG-BING WEI, DONG LIU, JUN-HUI YU, SHAO-YING LU, HUI WANG

**Affiliations:** 1 Department of General Surgery, First Affiliated Hospital of Medical College, Xi’an Jiaotong University, Xi’an, Shaanxi 710061, P.R. China; 2 Second Department of Cardiovascular Medicine, Shaanxi Provincial People’s Hospital, Xi’an, Shaanxi 710068, P.R. China; 3 Department of Anesthesiology, Shaanxi Provincial People’s Hospital, Xi’an, Shaanxi 710068, P.R. China

**Keywords:** hypoxia response element, human telomerase catalytic subunit, caudal-related homeobox protein 2, proliferation, colon cancer

## Abstract

Caudal-related homeobox protein 2 (CDX2), a tumor suppressor in the adult colon, is overexpressed under a non-cancer specific cytomegalovirus promoter in certain tumor cells; furthermore, non-specific expression of CDX2 may result in aberrant side effects in normal cells. The human telomerase reverse transcriptase (hTERT) promoter is active in the majority of cancer cells but not in normal cells. Hypoxia is a key feature of solid tumors, and targeted genes may be significantly upregulated by five copies of hypoxia-response elements (HREs) under hypoxic conditions. However, the effect of CDX2 overexpression, as controlled by five copies of HREs and the hTERT promoter, on human colorectal cancer (CRC) cell proliferation *in vitro* remains to be fully elucidated. In the current study, a recombinant lentivirus containing the CDX2 gene under the control of five HREs and the hTERT promoter was generated. An immunofluorescence assay was used to detect CDX2 expression by the 5HhC lentivirus, whereas an MTT assay was used to detect the effects of CoCl_2_ on the viability of LoVo cells. Western blot analysis was conducted in order to determine the relative ratios of recombinant CDX2 protein to the internal control β-actin, following 5HhC/LoVo cell culture under normoxic and hypoxic conditions (100, 200, 300, 400 or 500 *µ*mol/l CoCl_2_) for 24 h, then for 12, 24 or 36 h with the optimal concentration (300 *µ*mol/l) of CoCl_2_. Reverse transcription polymerase chain reaction analysis was used to determine the transcription of recombinant CDX2 mRNA following culture of 5HhC/LoVo cells under normoxic or hypoxic conditions. Finally, a cloning assay was used to detect the proliferative ability of 5HhC/LoVo and 5Hh cells. High CDX2 expression was observed in hTERT-positive LoVo cells under hypoxic conditions, an effect which was mimicked by treatment with CoCl_2_ to inhibit LoVo cell proliferation *in vitro*. High expression of CDX2 therefore provides a promising strategy for the development of novel targeted treatments and gene therapy for CRC.

## Introduction

Colorectal cancer (CRC) is the third most common type of cancer worldwide, with ~1.4 million novel cases diagnosed in 2012 (1). At present, the treatment of CRC involves surgical resection, chemotherapy, radiotherapy and immunotherapy (2). However, the therapeutic efficacy, particularly for advanced CRC, is limited (3).

Caudal-related homeobox protein 2 (CDX2), an intestinal transcription factor, is critically involved in the development, proliferation and differentiation of intestinal epithelial cells (4–7). In addition, CDX2 is a tumor-suppressor gene in colorectal cancer (8–10), and reduces mobility and antagonizes dissemination of colon cancer cells *in vitro* and *in vivo* (11). By contrast, the presence of reduced CDX2 expression levels is a predictor for poor overall survival amongst patients with colorectal cancer (12). Therefore, forced overexpression of CDX2 under a cytomegalovirus (CMV) promoter in colon cancer cells is used to inhibit LoVo colon cancer cell invasion (13) and gastric cancer progression (14).

However, due to the fact that non-specific expression of CDX2 may lead to the generation of side effects, regulated colorectal cancer cell-specific expression of CDX2 is necessary. The human telomerase reverse transcriptase (hTERT) promoter is active in the majority of cancer cells but not normal cells (15,16). Therefore, this promoter has previously been used to target A549 human lung adenocarcinoma cells (17) and human gastric cancer MKN45 cells (18).

Hypoxia is a major feature of solid tumors (19,20) and induces hypoxia-inducible factor-1α (HIF-1α) expression, which binds to the hypoxia-response elements (HREs) of various target genes (21) and activates their transcription in order to regulate glucose transport and angiogenesis, and potentially to enhance the survival of tumor cells (22,23). Previous studies have reported that targeted genes may be significantly upregulated by five copies of HREs under hypoxic conditions (24,25).

At present, the effects of CDX2 overexpression, under the control of five copies of HREs and the hTERT promoter, on human colorectal cancer cell proliferation *in vitro* remain unclear. In the current study it was hypothesized that CDX2 overexpression specifically inhibits human colorectal cancer cell proliferation under hypoxic conditions.

## Materials and methods

### Polymerase chain reaction (PCR) amplification of target DNA

The hTERT gene promoter and CDX2 gene were amplified from a DNA library of hTERT(+) CRC cells and pEGFP-C1-CDX2 (26), respectively, by PCR using specific primers (Table I). hTERT was obtained using the hTERT forward and reverse1 primer, whereas 5HRE + hTERT used the forward 5HRE primer and the hTERT reverse2 primer. For the hTERT promoter, the PCR cycling conditions were as follows: Amplification at 98°C for 2 min, 30 cycles of 98°C for 10 sec, 55°C for 15 sec and 72°C for 30 sec, followed by an extension step at 72°C for 10 min. For the CDX2 promoter, the conditions were as follows: Amplification at 98°C for 2 min, 35 cycles of 98°C for 20 sec, 59°C for 30 sec and 72°C for 1 min, followed by an extension step at 72°C for 10 min. This was performed using the PTC-100 (Bio-Rad Laboratories, Inc., Hercules, CA, USA).

The PCR products were resolved on a 1% agarose gel electrophoresis (Shaanxi Pioneer Biotech Co., Ltd., Xi’an, China). The hTERT promoter (p) and CDX2 products were digested with *Hin*dIII/*Pst*I and *Eco*RI/*Bam*HI, respectively, and confirmed by DNA sequence analysis at Sangon Biotech Co. Ltd. (Shanghai, China).

### Construction of lentiviral vectors

The hTERT promoter was first cloned into the pLenhanced green fluorescent protein (EGFP)-N1-5HRE-CEAp (27) plasmid (Translational Medical Center, First Affiliated Hospital of Xi’an Jiaotong University, Xi’an, China) at the *Hin*dIII and *Pst*I sites by replacing CEAp with the restriction endoenzymes for 16 h at 37°C, in order to derive a recombinant plasmid named pLEGFP-N1-5HRE-hTERTp. An incision enzyme and Taq DNA polymerase from Takara Bio, Inc. (Otsu, Japan) were used. Subsequently, the 5HRE-hTERTp fragment, which was digested with the restriction endoenzymes *Bg*III and *Pst*I in buffer at 37°C for 16 h, was cloned into the lentiviral vector pLVX-EGFP-3FLAG (Translational Medical Center, First Affiliated Hospital of Xi’an Jiaotong University) by replacing the CMV promoter in the plasmid to generate the recombinant plasmid pLVX-5HRE-hTERTp-EGFP-3FLAG (designated as 5Hh), into which the amplified CDX2 fragment was cloned by replacing EGFP to produce the recombinant plasmid pLVX-5HRE-hTERTp-CDX2-3FLAG (designated as 5HhC). The identity of the final recombinant lentiviral vector construct was confirmed by restriction endonuclease digestion and DNA sequence analysis at Sangon Biotech Co., Ltd.

### Cell lines and cell cultures

Human epithelial kidney HEK 293T, human proximal tubular HK-2 and human CRC LoVo cells (Translational Medical center, First Affiliated Hospital of Xi’an Jiaotong University) were cultured in Dulbecco’s modified Eagle’s medium (DMEM) from Gibco Life Technologies (Carlsbad, CA, USA), supplemented with 10% fetal bovine serum obtained from GE Healthcare Life Sciences (Logan, UT, USA) in a humidified atmosphere with 5% CO_2_ at 37°C.

### Generation and titration of lentiviruses

Recombinant lentiviruses were produced by co-transfecting 293T cells with the lentiviral expression plasmid 5HhC or the control plasmid 5Hh, in addition to the pCD/NL-BH*DDD and pLTR-G plasmids (Shanghai Sunbio Technology Co., Ltd., Shanghai, China) using Trans-EZ reagent (Shanghai Sunbio Technology Co., Ltd.). The 293T cells (6×10^5^) were cultured in a 10-cm tissue culture plate with opti-MEM (Gibco Life Technologies). Transfection was performed when the cell density reached 30–40% confluence. Solution A was prepared by adding 0.5 ml (0.5 mg/ml) 5HhC or 5Hh plasmid and 1 ml (0.2 mg/ml) pCD/NL-BH*DDD or 0.5 ml (0.2 mg/ml) pLTR-G plasmids (diluted with opti-MEM media) to 18 ml opti-MEM media. Solution B was prepared by adding 0.5 ml Trans-EZ to 18 ml opti-MEM media. The transfection solution was prepared by adding solution B slowly to solution A. The mixture was agitated and then incubated at room temperature for 20 min. The 293T cells were plated in six-well plates prior to incubation with 3 ml prepared transfection mixture in a humidified incubator with 5% CO_2_ at 37°C for 30 min. The culture media was then replaced with fresh DMEM. Infectious lentiviruses were harvested at 48 h post-transfection by collecting the medium and centrifuging at 500 × g and 4°C for 10 min, then filtering the supernatant containing the lentiviruses through a 0.45 *µ*m polyvinylidene fluoride filter unit (EMD Millipore, Billerica, MA, USA) to concentrate it. The infectious titer was determined by quantitative PCR (qPCR). The total RNA of the transfected cells was extracted using TRIzol Reagent (Invitrogen Life Technologies,Carlsbad, CA, USA). Reverse transcription and PCR were performed using a Takara Bio, Inc. RNA PCR (AMV) kit with the CDX2 primers presented in Table I. The results were normalized against the level of the internal control. β-actin PCR amplifications were performed at 98°C for 2 min, 40 cycles of 50°C for 2 min, 95°C for 10 min and 95°C for 15 sec, followed by an extension step at 60°C for 1 min (ABI PRISM 7000 Sequence Detection System). qPCR was performed in triplicate for each experiment, including for the non-template controls.

### Infection of LoVo cells with lentiviruses

LoVo cells were cultured in six-well plates (5×10^5^cells/well) overnight, infected with 5HhC or control 5Hh lentiviruses and exposed to puromycin (800 *µ*g/ml; Gibco Life Technologies) for two weeks. The 5HhC/LoVo or 5Hh/LoVo cells were then routinely cloned (28).

### Immunofluorescence assay

To detect CDX2 expression by the 5HhC lentivirus, the hTERT(+) LoVo and hTERT(-) HK-2 cells were infected with 5HhC lentiviruses in a humidified incubator with 5% CO_2_ at 37°C for 48 h and the CDX2 expression was examined using an immunofluorescence assay. The 5HhC-infected LoVo and HK-2 cells were cultured and fixed with 4% paraformaldehyde (Shaanxi Pioneer Biotech Co., Ltd.) for 30 min. The cells were stained with a primary monoclonal rabbit anti-FLAG antibody (1:200; F2555; Sigma-Aldrich, St. Louis, MO, USA) at 4°C overnight prior to incubation at 37°C for 30 min with a fluorescein isothiocyanate-conjugated goat anti-rabbit immunoglobulin (Ig) G secondary antibody (1:5,000; Wuhan Sanying Biotechnology, Wuhan, China). The cells were then mounted with DAPI solution (Santa Cruz Biotechnology, Inc., Dallas, TX, USA) and observed under a confocal fluorescence microscope (Leica DMi8; Leica Microsystems GmbH, Wetzlar, Germany) to evaluate FLAG expression.

### MTT assay

The effects of CoCl_2_ on the viability of LoVo cells were detected using an MTT assay. The LoVo cells, at a density of 5×10^3^cells/well, were cultured for 24 h and then treated with 100, 200, 300, 400 or 500 *µ*mol/l CoCl_2_ (Sigma-Aldrich) for 1, 3, 5 or 7 days. The experiment was performed in triplicate. Subsequently, the plates were washed extensively with serum-free DMEM to remove CoCl_2_ and dead cells, and were exposed to 20 *µ*l (5 g/l) MTT (Sigma-Aldrich) for 4 h. The resulting formazan crystals were dissolved in 200 *µ*l dimethyl sulfoxide (Sigma-Aldrich) and the absorbance was measured at 490 nm with a microplate reader (Victor3; PerkinElmer, Inc., Waltham, MA, USA). The cytotoxicity of CoCl_2_ to LoVo cells was evaluated by determination of the survival rate of LoVo cells (calculated as A490 of treatment group/A490 of untreated group).

### Western blot analysis

The 5HhC/LoVo cells were cultured under a normoxic (absence of CoCl_2_) or hypoxic conditions (100, 200, 300, 400 or 500 *µ*mol/l CoCl_2_) for 24 h and then cultured for 12, 24 or 36 h with the optimal concentration (300 *µ*mol/l) of CoCl_2_. The relative ratios of recombinant CDX2 protein to control β-actin were determined by western blot analysis. Briefly, the 5HhC lentivirus-infected LoVo cells (1×10^6^ cells) were lysed with 150 *µ*l lysis buffer (50 mM Tris, 150 mM NaCl, 5 mM EDTA, 5 mM EGTA and 1% SDS; pH 7.5; (Shaanxi Pioneer Biotech Co., Ltd.), followed by gentle sonication (Bio-Rad Laboratories, Inc.). Following quantification with Bradford reagent (Thermo Fisher Scientific, Waltham, MA, USA), the protein lysates (80 *µ*g/lane) of each sample were subjected to SDS-PAGE (Shaanxi Pioneer Biotech Co., Ltd.) with a machine from EMD Millipore on 10% gels and transferred to polyvinylidene fluoride membranes (EMD Millipore). Subsequent to being blocked with 5% fat-free dry milk, the membranes were incubated with 1:1,000 diluted monoclonal rabbit anti-CDX2 (2475-1; Epitomics, Burlingame, CA, USA) and 1:3,000 diluted polyclonal rabbit anti-β-actin (BS10005; Bioworld Technology, Inc., St. Louis Park, MN, USA) at 4°C, overnight prior to incubation with horseradish peroxidase-conjugated goat anti-rabbit IgG secondary antibody (OriGene Technologies, Inc., Beijing, China) for 30 min at 37°C, followed by visualization with enhanced chemiluminescence (Amresco LLC, Solon, OH, USA).

### Reverse transcription (RT)-PCR analysis

The 5HhC/LoVo cells were cultured under normoxic or hypoxic conditions and the transcription of recombinant CDX2 mRNA was determined by RT-PCR using the primers listed in Table I. In brief, total RNA was extracted from the infected cells cultured under normoxia or hypoxia using TRIzol (Gibco Life Technologies, Grand Island, NY, USA), according to the manufacturer’s instructions. The resulting RNAs were treated with RNase-free DNase (Promega Corp., Madison, WI, USA) and reverse transcribed into cDNA using an RT-PCR kit (Invitrogen Life Technologies) according to the manufacturer’s instructions. PCR amplifications were performed in duplicate at 98°C for 2 min and subjected to 35 cycles of 98°C for 20 sec, 59°C for 30 sec and 72°C for 1 min, followed by an extension at 72°C for 10 min. The PCR products were resolved by agarose gel electrophoresis using a ChemiDoc System (ChemiDoc™ MP System 170-8280; Bio-Rad Laboratories, Inc.).

### Cloning assay

A total of 200 5HhC/LoVo and 200 5Hh cells plated on 60-mm cell culture dishes were cultured in a humidified incubator with 5% CO_2_ at 37°C for three weeks. The cloning cells were fixed with methanol (Shaanxi Pioneer Biotech Co., Ltd.) at room temperature for 15 min and then stained by Giemsa solution. The clones containing ≥50 cells [counted using a Leica M125 microscope (Leica Microsystems GmbH, Heidelberg, Germany)] were regarded as true clones (29).

### Statistical analysis

All experiments were repeated three times. The data from all experiments was pooled, and the results were expressed as the mean ± standard deviation. Differences in mean values were analyzed by one-way analysis of variance followed by Student’s t-test with SPSS software version 13.0 (SPSS, Inc., Chicago, IL, USA). P<0.05 was considered to indicate a statistically significant difference.

## Results

### Recombinant plasmids were successfully generated

**Figure 1:**
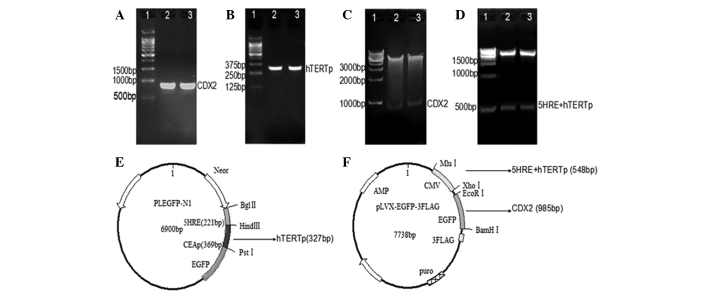
Characterization of recombinant plasmids. (A) Electrophoretogram of CDX2 PCR products. (B) Electrophoretogram of hTERTp PCR products. (C) Restriction endonuclease enzyme digestions of pLVX-5HRE-hTERTp-CDX2-3FLAG DNA. (D) Restriction endonuclease enzyme digestions of pLEGFP-N1-5HRE-hTERTp DNA. Lanes: 1, DNA marker; 2 and 3, PCR or enzyme-digested products. (E) Schematic chart of the recombinant plasmid pLEGFP-N1-5HRE-hTERTp. (F) Schematic chart of the recombinant plasmid pLVX-5HRE-hTERTp-CDX2-3FLAG. CDX2, caudal-related homeobox; PCR, polymerase chain reaction; hTERTp, human telomerase reverse transcriptase promoter; bp, base pairs.

The 327 base pairs (bp) hTERTp and 985 bp CDX2 DNA fragments were successfully amplified by PCR (Fig. 1A and B). The restricted enzyme digestions (Fig. 1C and D) and sequencing demonstrated that the recombinant plasmids pLEGFP-N1-5HRE-hTERTp and pLVX-5HRE-hTERTp-CDX2-3FLAG (Fig. 1E and F) were also successfully constructed.

### 5HhC-infected LoVo cells, but not HK-2 cells, express FLAG in vitro

**Figure 2:**
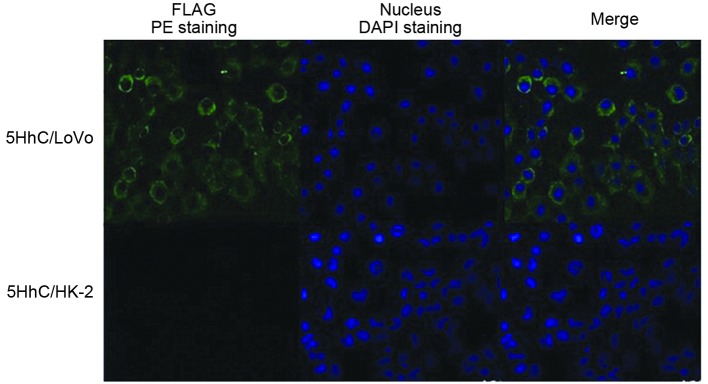
5HhC-infected LoVo cells, but not HK-2 cells, are positive for immunofluorescent FLAG staining. The 5HhC-infected LoVo and HK-2 cells were fixed with 4% paraformaldehyde for 30 min and stained with monoclonal rabbit anti-FLAG antibody and fluorescein isothiocyanate-goat anti-rabbit immunoglobulin G, mounted with DAPI solution and observed under a fluorescence microscope. Data presented are representative images (magnification, x100) from two separate experiments. 5HhC, pLVX-5HRE-hTERTp-CDX2-3FLAG; PE, plasmalemma.

An immunofluorescence assay was conducted to examine FLAG expression of 5HhC in the hTERT(+) LoVo and hTERT(−) HK-2 cell lines. It was observed that FLAG was expressed on the membrane and cytoplasm of 5HhC-infected LoVo cells, but not 5HhC-infected HK-2 cells (Fig. 2). This indicated that hTERT specifically directed exogenous gene expression in hTERT(+), but not hTERT(−) cell lines.

### High concentrations of CoCl_2_ attenuate LoVo cell proliferation in vitro

**Figure 3:**
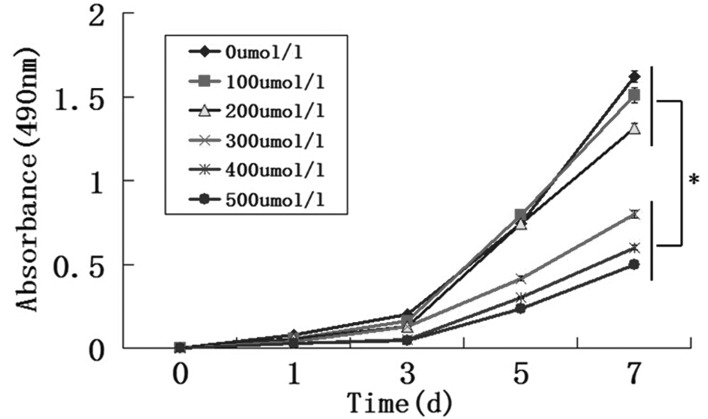
High concentrations of CoCl_2_ inhibit the proliferation of LoVo cells *in vitro*, determined by MTT assay. LoVo cells were treated with various concentrations of CoCl_2_ (100-500 *µ*mol/l) for seven days. A total of six samples were analyzed in each group. The assay was repeated independently a minimum of three times. Survival rate (%)=1-treatment group A490 nm/untreated group A490 nm. Values are presented as the mean ± standard deviation; ^*^P<0.05.

The cytotoxic effect of CoCl_2_ on LoVo cells was investigated using an MTT assay. The results demonstrated that cell viability was not influenced by CoCl_2_ at 100 or 200 *µ*mol/l (P>0.05), but the cell viability was suppressed with 300–500 *µ*mol/l CoCl_2_ (P<0.05), particularly at day 7 (Fig. 3).

### High CDX2 expression levels with 5HhC are induced in the hTERT(+) cell line under hypoxic conditions

**Figure 4:**
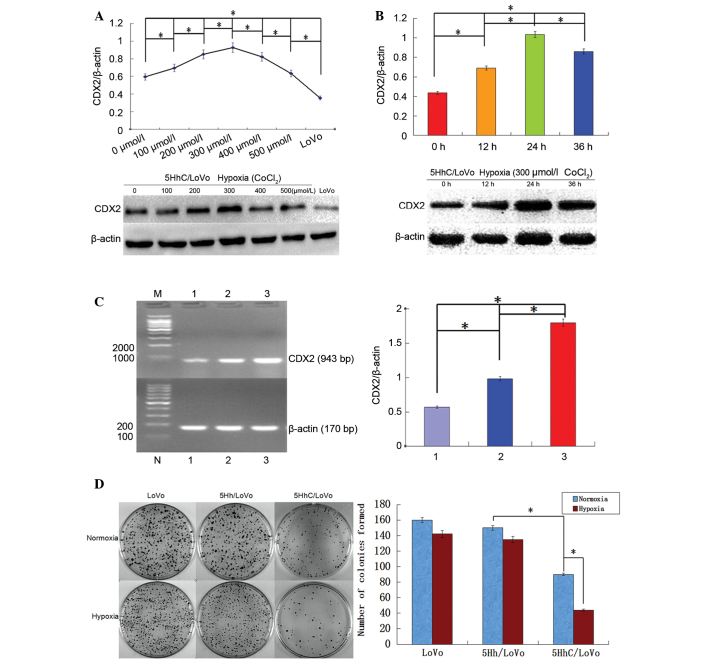
Expression levels of CDX2 in 5HhC LoVo cells and the effect of CDX2 overexpression on LoVo cell proliferation. (A) Western blot analysis of CDX2 expression. The 5HhC/LoVo cells and LoVo cells were cultured under normoxic or hypoxic conditions (100–500 *µ*mol/l CoCl_2_) for 24 h. (B) Western blot analysis of CDX2 expression. The 5HhC/LoVo cells were cultured under hypoxic conditions (300 *µ*mol/l CoCl_2_) for 0, 12, 24 or 36 h. (C) Reverse transcription-polymerase chain reaction analysis of CDX2 mRNA expression. The 5HhC/LoVo cells were cultured under a normoxic or hypoxic conditions (300 *µ*mol/l CoCl_2_) for 24 h. M, DNA marker; 1, LoVo cells; 2, 5HhC/LoVo cells under normoxic conditions; 3, 5HhC/LoVo cells under hypoxic conditions. (D) Clone formation of LoVo, 5Hh/LoVo or 5HhC/LoVo cells. Each group had a hypoxic control (200 *µ*mol/l CoCl_2_; red bars). All data presented are representative images of each group of cells from three separate experiments. The results are presented as the mean ± standard deviation (^*^P<0.05). CDX2, caudal-related homeobox; 5HhC, pLVX-5HRE-hTERTp-CDX2-3FLAG; 5Hh, pLVX-5HRE-hTERTp-EGFP-3FLAG; bp, base pairs.

The levels of recombinant CDX2 protein relative to control β-actin expression following CoCl_2_ hypoxic treatment over time was compared between 5HhC/LoVo and LoVo cells. Following 24 h of hypoxic treatment, the relative expression levels of CDX2 were upregulated at CoCl_2_ concentrations from 100–500 *µ*mol/l (P<0.05), with the greatest effect on CDX2 expression observed following treatment with 300 *µ*mol/l CoCl_2_, compared with that of 5HhC/LoVo cells without CoCl_2_ treatment (P<0.05; Fig. 4A). In 5HhC/LoVo cells treated with 300 *µ*mol/l CoCl_2_, the highest CDX2 expression levels were observed following 24 h of treatment when compared with those at 0, 12 or 36 h (P<0.05; Fig. 4B). Subsequently, 5HhC/LoVo cells were treated for 24 h under normoxic or hypoxic conditions (with 300 *µ*mol/l CoCl_2_). The relative expression levels of CDX2 mRNA in 5HhC/LoVo cells under hypoxic conditions were observed to be significantly higher when compared with those under normoxic conditions (P<0.05; Fig. 4C). The data presented indicated that the CDX2 mRNA and protein expression levels were upregulated in 5HhC/LoVo cells, particularly under hypoxic conditions.

To further assess the potential effects of CDX2 expression with 5HhC on the proliferation of LoVo cells, a cloning assay was conducted with 5HhC/LoVo and LoVo cells. The proliferation results demonstrated that the clone numbers and size in 5HhC/LoVo cells were significantly reduced compared with those of LoVo cells (P<0.05). Notably, the proliferation levels of 5HhC/LoVo cells under hypoxic conditions were significantly lower compared with those under normoxic conditions (P<0.05; Fig. 4D). These results indicated that a hypoxic environment resulted in upregulation of CDX2 expression with 5HhC to inhibit LoVo cell proliferation via the 5 HRE enhancers.

## Discussion

In the present study, a recombinant lentivirus with enhanced CDX2 expression driven by hTERTp and 5 HRE enhancers was generated, and it was observed that CDX2 was highly expressed in hTERT positive cells under hypoxic conditions, which attenuated CRC cell proliferation *in vitro*.

It was identified that CDX2 expression was detected in hTERT(+) LoVo cells, but not in hTERT(−) HK-2 cells, suggesting that the 5HhC vector was hTERT-specific *in vitro*. The hTERT promoter, which is active in the majority of cancer cell lines but not in normal cells, has been previously reported as a useful tool for tumor transcriptional targeting (30–32). In addition, it has been demonstrated to effectively target A549 human lung adenocarcinoma cells and MKN45 human gastric cancer cells (16).

In order to enhance CDX2 expression under the hTERT promoter, five copies of HRE were positioned upstream of the hTERT promoter. It was observed that 5HhC/LoVo cells under hypoxic conditions, produced by treatment with 300 *µ*mol/l CoCl_2_ for 24 h, exhibited the greatest expression levels of CDX2 compared with those under normoxic conditions without CoCl_2_. A previous study reported that the development of CRC was dependent on the tumor microenvironment (33). The observations of the current study were in agreement with a previous study, in which five copies of HRE under the control of a CMV promoter induced bacterial cytosine deaminase expression in the bacterial cytosine deaminase/5-fluorocytosine gene therapy system under hypoxic conditions, enhancing the efficacy of radiotherapy in a tumor xenograft (34). In the present study, it was observed that 5HhC/LoVo cells under hypoxic conditions exhibited significantly inhibited LoVo cell proliferation compared with those under normoxic conditions, suggesting that CDX2 expression was induced under hypoxic conditions to suppress CRC cell proliferation. This is consistent with the role of CDX2 as a tumor-suppressor gene in colorectal cancer (35). A non-transcriptional function of CDX2 is that it suppresses tumorigenesis via p27Kip1 stabilization, and low levels of CDX2 have been reported to accelerate colon tumorigenesis by reducing p27Kip1 levels (36). Notably, mutation of a subdomain in the N-terminus of CDX2 has been reported to abrogate the anti-proliferative effects of CDX2, which may be via inhibition of β-catenin/T-cell factor (TCF) transcriptional activity by disrupting the β-catenin-TCF protein complex in colon cancer cells (37). CDX2 tumor suppression may also function via the extracellular signal-regulated kinase 1/2 pathway in CRC (38). Clinically, a reduction in CDX2 expression is correlated with poor overall survival amongst patients with colorectal cancer (39).

In conclusion, a recombinant lentivirus with the hTERT promoter and five HREs promoted expression of the tumor suppressor CDX2 in CRC cells under hypoxic conditions, and was effective at targeting CRC cells *in vitro*. CDX2 expression under the hTERT promoter and five HREs may provide a potential tool for the treatment and gene therapy of CRC.

## Figures and Tables

**Table I:** Primer sequences.

Gene	Primer	Base sequence 5′-3′	PCR product (base pairs)
5HRE	Forward	CGACGCGTATTATGCTAGTCCAC	221
hTERT	Forward	CCCAAGCTTCACAGACGCCCAGGACCGCGCTTC	327
	Reverse1	AACTGCAGCCACGTGCGCCCACGTGCGCCCAC	
	Reverse2	CCGCTCGAGCCACGTGCGCCCACGTGCGCCCAC	
CDX2	Forward	CGGAATTCATGTACGTGAGCTACCTCCTGGACAAGGAC	943
	Reverse	CGGGATCCGTCTGGGTGACGGTGGGGTTTAGCACCCCCCCAGTTG	

Underlined sections indicate the restriction sites. PCR, polymerase chain reaction.

## Abbreviations

CDX2: caudal-related homeobox protein 2
hTERT: human telomerase reverse transcriptase
HREs: hypoxia-response elements
CMV: cytomegalovirus
CRC: colorectal cancer
MTT: 3-(4,5-dimethylthiazol-2yl)-2,5-diphenyltetrazolium bromide
